# Unique Roles for *Streptococcus pneumoniae* Phosphodiesterase 2 in Cyclic di-AMP Catabolism and Macrophage Responses

**DOI:** 10.3389/fimmu.2020.00554

**Published:** 2020-03-31

**Authors:** Alicia K. Wooten, Anukul T. Shenoy, Emad I. Arafa, Hisashi Akiyama, Ian M. C. Martin, Matthew R. Jones, Lee J. Quinton, Suryaram Gummuluru, Guangchun Bai, Joseph P. Mizgerd

**Affiliations:** ^1^Pulmonary Center, Boston University School of Medicine, Boston, MA, United States; ^2^Department of Medicine, Boston University School of Medicine, Boston, MA, United States; ^3^Department of Microbiology, Boston University School of Medicine, Boston, MA, United States; ^4^Department of Pathology and Laboratory Medicine, Boston University School of Medicine, Boston, MA, United States; ^5^Department of Immunology and Microbial Disease, Albany Medical College, Albany, NY, United States; ^6^Department of Biochemistry, Boston University School of Medicine, Boston, MA, United States

**Keywords:** cyclic di-AMP, innate immunity, interferon-β, macrophages, pneumococcus, pneumonia, STING

## Abstract

Cyclic di-AMP (c-di-AMP) is an important signaling molecule for pneumococci, and as a uniquely prokaryotic product it can be recognized by mammalian cells as a danger signal that triggers innate immunity. Roles of c-di-AMP in directing host responses during pneumococcal infection are only beginning to be defined. We hypothesized that pneumococci with defective c-di-AMP catabolism due to phosphodiesterase deletions could illuminate roles of c-di-AMP in mediating host responses to pneumococcal infection. Pneumococci deficient in phosphodiesterase 2 (Pde2) stimulated a rapid induction of interferon β (IFNβ) expression that was exaggerated in comparison to that induced by wild type (WT) bacteria or bacteria deficient in phosphodiesterase 1. This IFNβ burst was elicited in mouse and human macrophage-like cell lines as well as in primary alveolar macrophages collected from mice with pneumococcal pneumonia. Macrophage hyperactivation by Pde2-deficient pneumococci led to rapid cell death. STING and cGAS were essential for the excessive IFNβ induction, which also required phagocytosis of bacteria and triggered the phosphorylation of IRF3 and IRF7 transcription factors. The select effects of Pde2 deletion were products of a unique role of this enzyme in c-di-AMP catabolism when pneumococci were grown on solid substrate conditions designed to enhance virulence. Because pneumococci with elevated c-di-AMP drive aberrant innate immune responses from macrophages involving hyperactivation of STING, excessive IFNβ expression, and rapid cytotoxicity, we surmise that c-di-AMP is pivotal for directing innate immunity and host-pathogen interactions during pneumococcal pneumonia.

## Introduction

Pneumonia is a leading cause of morbidity and mortality worldwide (1). Children under 5 and adults over 60 years of age have the greatest risk of developing pneumonia (2). The most common bacterial cause of community acquired pneumonia is the gram-positive extracellular bacterium, *Streptococcus pneumoniae* (pneumococcus). Pneumococcus can colonize the nasopharynx asymptomatically, or it can cause diseases such as otitis media, bacteremia, or meningitis, in addition to pneumonia (3). Despite advances with pneumococcal vaccines, infections with non-vaccine serotypes remain important (4). A better understanding of the pathogen and the host immune response may help develop novel prophylactics and therapeutics to lower the disease burden caused by *S. pneumoniae*.

In the lungs, alveolar macrophages are among the first lines of immune defense (5). Multiple receptors on the surface of macrophages recognize bacteria, leading to phagocytosis and the expression of cytokines and chemokines. During pneumococcal pneumonia, NF-κB activation in macrophages is required to rapidly ramp up cytokine expression andneutrophil recruitment (6). Cytokines such as TNFα, IL-1, CXCL2, and IFNβ are produced by macrophages to elicit lung immunity (5). TNFα, IL-1, and CXCL2 all contribute to neutrophil-mediated killing of pneumococcus in the lungs (7, 8). IFNβ and its receptor IFNAR1 help mediate resistance against pneumococcal infection by decreasing nasal colonization (9), enhancing compartmentalization of bacteria within the infected respiratory tract (10), and increasing lung epithelial cell resilience against the infectious and inflammatory challenges of pneumonia (11). However, excessive or inappropriate type I interferon signaling compromises immune defense and predisposes the host to pneumococcal pneumonia (12–14). The induction of IFNβ by pneumococcus is mediated via a pathway in which the endoplasmic reticulum transmembrane protein STimulator of Interferon Genes (STING) triggers the TANK Binding Kinase 1 (TBK1) to activate the transcription factor interferon regulatory factor 3 (IRF3) (9, 15). STING is an adaptor molecule and receptor for cyclic dinucleotides in the cytosol including the bacterial metabolites cyclic di-GMP and cyclic di-AMP (c-di-AMP), as well as the mammalian-derived cyclic GMP-AMP (cGAMP) produced by the cyclic GMP-AMP synthase (cGAS) enzyme (16–18). Induction of IFNβ by pneumococcus requires both STING and cGAS (19), which was interpreted as evidence that pneumococcal DNA but not pneumococcal c-di-AMP triggers STING-dependent IFNβ induction (19). However, a cGAS-STING complex is essential to IFNβ induction by purified c-di-AMP (20), which could contribute to the requirement for cGAS in pneumococcus-induced signaling. Specific effects of pneumococcal c-di-AMP on macrophage responses remain to be demonstrated.

The roles of c-di-AMP in bacteria are diverse and include potassium transport, resistance to cell wall stress, peptidoglycan homeostasis, biofilm formation, and modulating the cellular size of the bacteria (21–25). Pneumococcus generates c-di-AMP using a diadenylate cyclase, CdaA (26). c-di-AMP is degraded in pneumococcus by two phosphodiesterase enzymes, Phosphodiesterase 1 (Pde1) and Pde2 (26). Pde1 converts c-di-AMP to 5′-phosphoadenylyl-adenosine (pApA), while Pde2 is capable of catabolizing both c-di-AMP and pApA, in each case resulting in AMP as a product. Deletion of one or both of the phosphodiesterase enzymes impairs c-di-AMP catabolism, increasing its concentrations in the mutant pneumococci (26). The effects of pneumococcal c-di-AMP metabolism on host-pathogen interactions are largely unclear. We hypothesized that pneumococci deficient in the phosphodiesterase enzymes degrading c-di-AMP could illuminate macrophage pathways involved in innate immune responses to bacterial cyclic dinucleotides during infection.

## Results

### Elevated Macrophage NF-κB Responses to Phosphodiesterase-Mutant Pneumococci

**FIGURE 1:**
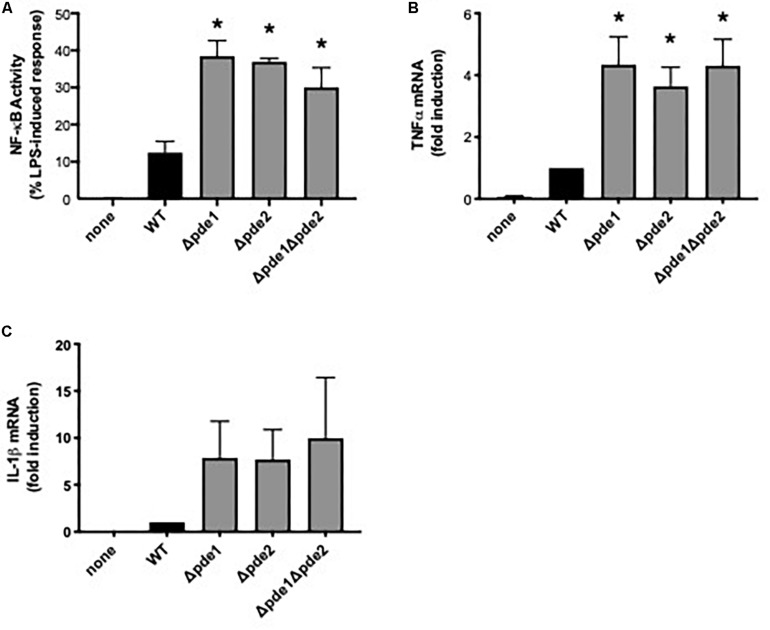
Phosphodiesterase mutations increase pneumococcus-induced NF-κB activity. **(A)** Relative NF-κB-mediated gene expression was measured using a mouse macrophage-like RAW264.7 cell line which had been stably transduced with a firefly luciferase transgene responsive to NF-κB. Cultures were infected 2 h with the indicated bacteria, and luciferase values were quantified using a luminometer and expressed relative to LPS positive control wells run in parallel (*N* = 9 experiments). **(B)** Relative induction of TNFα mRNA was measured in RAW264.7 cell cultures infected 2 h with the indicated bacteria. TNFα mRNA was measured and normalized to 18S rRNA using qPCR, and expressed relative to the cells infected by WT bacteria (*N* = 3 experiments). **(C)** Relative induction of IL-1β mRNA was measured in RAW264.7 cell cultures infected 2 h with the indicated bacteria. IL-1β mRNA was measured and normalized to 18S rRNA using qPCR, and expressed relative to the cells infected by WT bacteria (*N* = 3 experiments). Throughout panels, asterisk (*) indicates *P* < 0.05 compared to WT.

Macrophage NF-κB is important to pneumococcal infection of the lung. During pneumococcal pneumonia, NF-κB RelA in macrophages is crucial for early cytokine expression, neutrophil recruitment, and lung defense (6). Macrophage activation of NF-κB is heterogeneous across clinical isolates, and lower NF-κB activators cause more severe lung infection in mouse models and associate with more severe pneumonia in human patients (27). To determine whether variations in c-di-AMP levels may influence the magnitude of macrophage NF-κB activation, we compared macrophage responses to 4 pneumococcal strains: a wild type (WT) strain and strains in which one (Δ*pde1* or Δ*pde2*) or both (Δ*pde1*Δ*pde2*) c-di-AMP-catabolizing enzymes were deleted, all in the isogenic D39 genetic background (26). Macrophage NF-κB activity was quantified using a mouse macrophage-like RAW264.7 cell line that was stably transduced to express firefly luciferase driven by tandem NF-κB binding sites (27, 28). All three pneumococcal phosphodiesterase mutants significantly increased NF-κB activity compared to the WT strain (Figure 1A). The different mutant strains had similar effects on macrophage NF-κB activation (Figure 1A). To determine if this influenced cytokine expression, we quantified TNFα and IL-1β induction. TNFα mRNA levels were significantly elevated after infection with the phosphodiesterase-deficient pneumococci, with comparable effects noted for all the mutant bacteria (Figure 1B). At the protein level, TNFα was elevated in cell culture supernatants which included bacteria, with those containing Δ*pde2* or Δ*pde1*Δ*pde2* pneumococci reaching statistical significance (mean ± SEM concentrations of 1790 ± 264, 2390 ± 157, 2370 ± 173, 2580 ± 108, and 2620 ± 37 in cultures containing no, WT, Δ*pde1*, Δ*pde2*, or Δ*pde1*Δ*pde2* pneumococci, respectively, from *N* = 3 independent experiments; *P* values compared to the no pneumococci group of 0.11, 0.12, 0.03, and 0.02, respectively, using one-way ANOVA and the Sidak *post hoc* test). IL-1β mRNA induction showed a similar trend but had more variability and did not reach statistical significance (Figure 1C). IL-1β protein was not detectable by ELISA in any culture supernatants at this 2-h time-point. Altogether, these data reveal that phosphodiesterase enzymes are determinants of macrophage innate immune responses to pneumococcus, and loss of the pneumococcal enzymes that catabolize c-di-AMP increases NF-κB activity.

### Mixed Macrophage IFNβ Responses to the Phosphodiesterase Mutants

**FIGURE 2:**
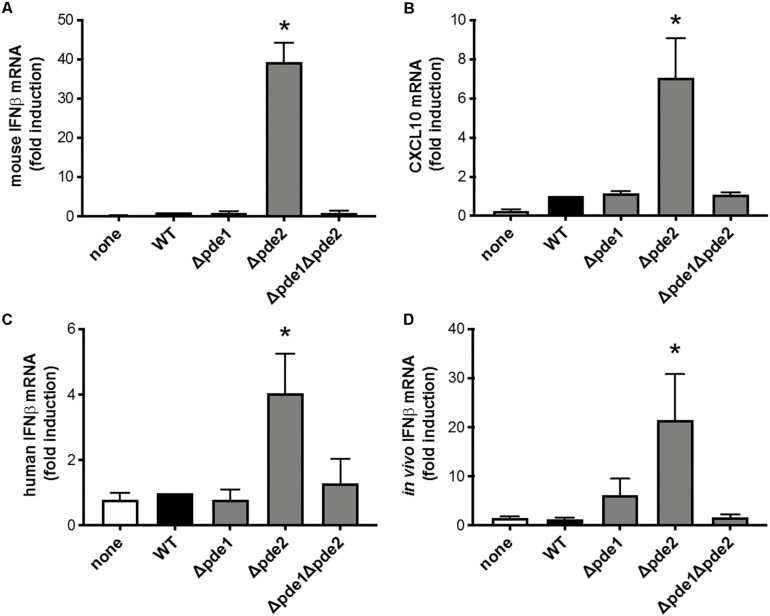
Phosphodiesterase 2 mutation in pneumococcus elicits a distinctive burst of IFNβ expression from macrophages. **(A)** Relative induction of IFNβ mRNA was measured in mouse macrophage-like RAW264.7 cell cultures infected 2 h with the indicated bacteria. Mouse IFNβ mRNA was measured and normalized to 18S rRNA using qPCR, and expressed relative to the cells infected by WT bacteria (*N* = 3 experiments). **(B)** Relative induction of mouse CXCL10 mRNA was measured and normalized to 18S rRNA using qPCR, and expressed relative to the cells infected by WT bacteria (*N* = 3 experiments). **(C)** Relative induction of IFNβ mRNA was measured in human THP-1 cell cultures that were matured to macrophage-like phenotypes using PMA before being infected 2 h with the indicated bacteria. Human IFNβ mRNA was measured and normalized to 18S rRNA using qPCR, and expressed relative to the cells infected by WT bacteria (*N* = 6 experiments). **(D)** Relative induction of IFNβ mRNA was measured in mouse alveolar macrophages collected by bronchoalveolar lavage 3 h after *in vivo* infection of mice by intratracheal instillation with the indicated bacteria. Mouse IFNβ mRNA in these cells collected from infected lungs was measured and normalized to 18S rRNA using qPCR, and expressed relative to the cells collected from mice infected by WT bacteria (*N* = 11–16 mice per group, over 4 experiments). Throughout panels, asterisk (*) indicates *P* < 0.05 compared to WT.

The innate immune response most directly responsive to c-di-AMP is type I IFN induction (29). To investigate if pneumococcal c-di-AMP could be a modulator of type I interferon responses, RAW264.7 macrophage-like cells were infected with the phosphodiesterase mutants and mRNA was collected so that IFNβ expression could be measured. We expected one of three outcomes: consistently elevated IFNβ responses across the mutant pneumococci, matching the observations with NF-κB activity (Figure 1); a ramping up of IFNβ responses with peak levels after infection by the double mutant Δ*pde1*Δ*pde2* strain, based on how c-di-AMP content in pneumococci increase due to phosphodiesterase mutations (26); or no effect of phosphodiesterase mutation, based on the postulate that pneumococcal DNA but not c-di-AMP drives IFNβ responses (19). Surprisingly, macrophage IFNβ responses matched none of these expectations. Instead, only macrophages infected with the Δ*pde2* mutant bacteria displayed enhanced levels of IFNβ after 2 h of infection (Figure 2A). In contrast to Δ*pde2* pneumococci, neither Δ*pde1* nor Δ*pde1Δpde2* pneumococci stimulated this burst of type I IFN expression. To determine whether this was a peculiarity of IFNβ alone, we measured another gene in this activation pathway. CXCL10 is also transcribed by the IRF3 transcription factor which STING activates (30, 31). Like IFNβ, CXCL10 expression was only elevated by phosphodiesterase mutation when *pde2* alone was targeted, not after infection with the Δ*pde1* or Δ*pde1Δpde2* pneumococci (Figure 2B). These results suggest, for the first time to our knowledge, that the two phosphodiesterase enzymes in *S. pneumoniae* have distinct and non-overlapping roles relating to infection and immunity.

In order to determine whether these results were robust and generalizable, we tested for macrophage IFNβ induction using a cell line from a different species (human instead of mouse) and using primary cells during an *in vivo* infection (alveolar macrophages collected from pneumonic lungs, instead of the RAW264.7 cell line in culture). Although the magnitude of response from human THP-1 cells was modest compared to mouse RAW264.7 cells, the relative effects of the different bacterial strains were similar across the different cell lines and species, showing increased IFNβ induction in response to Δ*pde2* bacteria compared to any and all other bacterial strains examined (Figure 2C). Thus, human and mouse cells share this distinctive response to Δ*pde2* mutant pneumococci. Mice were infected with pneumococci in their lungs, and bronchoalveolar lavage cells were collected after 3 h of *in vivo* infection (before neutrophil recruitment into the air spaces). IFNβ expression was measured in these cells using qRT-PCR, and the Δ*pde2* mutants showed significantly greater IFNβ induction than any of the other strains, which all behaved similar to each other (Figure 2D). Thus, the selective effects of mutation in phosphodiesterase 2 extend beyond *in vitro* cell lines and apply to primary alveolar macrophages from pneumonia experiments *in vivo*.

### Outcome of Infection With the Phosphodiesterase Mutants

**FIGURE 3:**
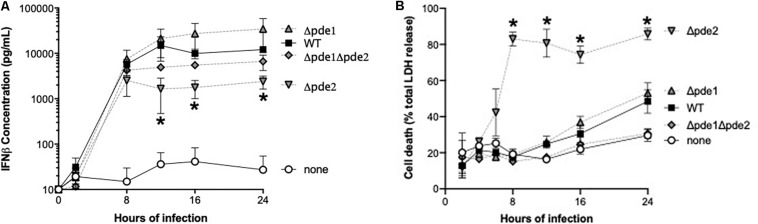
Outcome of infection is dictated by pneumococcal phosphodiesterase 2. **(A)** Time-course of IFNβ protein accumulation in the supernatant was measured in mouse macrophage-like RAW264.7 cell cultures infected with the indicated bacteria. Mouse IFNβ concentration was measured using ELISA (*N* = 3 experiments). **(B)** Time-course of cell death was measured in mouse macrophage-like RAW264.7 cell cultures infected with the indicated bacteria. Concentration of the cytosolic enzyme lactate dehydrogenase (LDH) was measured using a commercially available cytotoxicity assay kit, and expressed relative to concentrations measured after cells were completely lysed by detergent (*N* = 3 experiments). Throughout panels, asterisk (*) indicates *P* < 0.05 compared to WT.

We measured IFNβ protein in supernatants from infected RAW264.7 cells, expecting to see increased cytokine accumulation in cultures of the Δ*pde2* mutant infections compared to other bacteria. Cultures included living bacteria for 2 h, after which antibiotics wree provided to restore sterility to the cell cultures. All the bacteria stimulated IFNβ production, but over time the Δ*pde2* mutant infections stimulated significantly less IFNβ compared to the WT pneumococci (Figure 3A). This protein result after 12 or more hours of infection was the opposite of the early mRNA induction measured after 2–3 h of infection (Figure 2). This led us to question whether macrophage cultures infected with the Δ*pde2* mutant bacteria might deteriorate over time. We assessed macrophage viability after stimulation with the different strains of pneumococci using an LDH assay for cellular necrosis (Figure 3B). Again, there was a unique phenotype in macrophages infected with phosphodiesterase 2 mutant bacteria. By 8 h, more than 80% of the cultured cells were already dead when infected with the Δ*pde2* bacteria, whereas other strains exhibited only limited cytotoxicity at this time-point. Thus, the unique phenotypic responses of macrophages to Δ*pde2* mutant pneumococci includes rapid cytotoxicity. We therefore focused attention on early responses to infection with these mutants.

### Signaling Requirements for Exaggerated IFN Responses From the Phosphodiesterase Mutants

**FIGURE 4:**
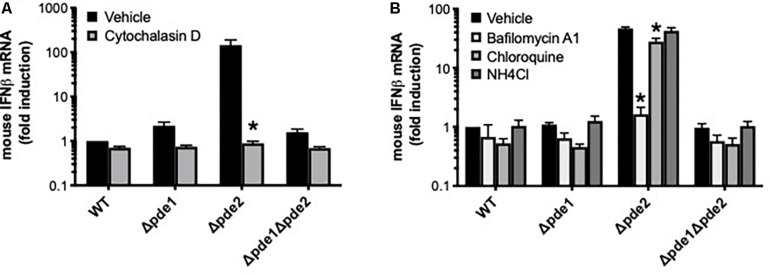
The induction of IFNβ by phosphodiesterase 2 mutant pneumococci requires phagocytosis. **(A)** Relative induction of IFNβ mRNA was measured in mouse macrophage-like RAW264.7 cell cultures infected 2 h with the indicated bacteria in the presence or absence of cytochalasin D as a means of inhibiting actin polymerization (*N* = 3 experiments). **(B)** Relative induction of IFNβ mRNA was measured in mouse macrophage-like RAW264.7 cell cultures infected 2 h with the indicated bacteria in the presence or absence of the indicated inhibitors pf phagosomal processing (*N* = 3 experiments). For both panels, mouse IFNβ mRNA was measured and normalized to 18S rRNA using qPCR, and expressed relative to the cells infected by WT bacteria. For both panels, asterisk (*) indicates *P* < 0.05 compared to vehicle.

We endeavored to elucidate the pathway by which Δ*pde2* mutant bacteria led to an elevated IFNβ burst. To determine if phagocytic uptake is involved, RAW264.7 cells were pretreated with the actin polymerization inhibitor cytochalasin D before infection with pneumococcus. Inhibition of actin polymerization prevented the burst of IFNβ expression after 2 h of infection by the Δ*pde2* mutant bacteria (Figure 4A). All strains, particularly the Δ*pde2* mutant, had diminished IFNβ expression, suggesting that phagocytosis is essential for the rapid and strong IFNβ induction by *pde2*-deficient bacteria. To determine roles of phagolysosomal fusion and acidification, we pretreated RAW264.7 cells with phagolysosome inhibitors. Bafilomycin A1, which inhibits vacuolar H^+^-ATPases and disrupts phagolysosome acidification and fusion, completely prevented IFNβ induction by the Δ*pde2* mutant bacteria (Figure 4B). Chloroquine and ammonium chloride (NH_4_Cl), lysosomotropic agents that can raise vacuolar pH without affecting fusion events, had more modest effects (Figure 4B). The profound effects of cytochalasin D and bafilomycin A1 on IFNβ induction suggest that Δ*pde2* mutant pneumococci stimulate macrophages after their phagocytosis and phagosomal processing.

**FIGURE 5:**
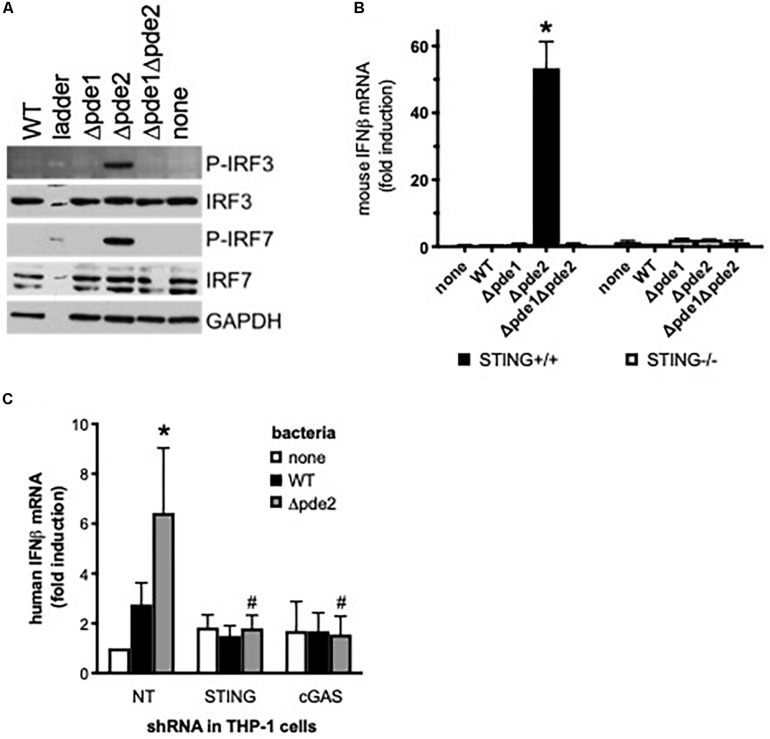
IFNβ expression elicited by phosphodiesterase 2 mutant pneumococci is mediated by the STING-cGAS-IRF3-IRF7 signaling pathway. **(A)** Relative contents of phosphorylated IRF3 and phosphorylated IRF7 protein were measured using immunoblot in cell lysates from mouse macrophage-like RAW264.7 cell cultures infected 2 h with the indicated bacteria. Relative contents of total IRF3 and IRF7 were measured for comparison, as well as GAPDH for loading control (image shown was from one experiment, representative of 2 independent experiments). **(B)** Relative induction of IFNβ mRNA was measured in parallel cultures of mouse macrophage-like RAW264.7 cells, one of which was deficient in STING due to gene targeting. Cultures were infected for 2 h with the indicated bacteria. Mouse IFNβ mRNA was measured and normalized to 18S rRNA using qPCR, and expressed relative to the cells infected by WT bacteria (*N* = 3 experiments). Asterisk (*) indicates *P* < 0.05 compared to WT. **(C)** Relative induction of IFNβ mRNA was measured in parallel cultures of human THP-1 cell cultures that were stably transduced with lentiviruses encoding shRNA that was non-targeting (NT) or which targeted STING or cGAS. THP-1 cells were matured to macrophage-like phenotypes using PMA before being infected 2 h with the indicated bacteria. Human IFNβ mRNA was measured and normalized to 18S rRNA using qPCR, and expressed relative to the uninfected cells transduced with NT shRNA (*N* = 5 experiments). Asterisk (*) indicates *P* < 0.05 compared to WT bacteria in cells transduced by the same shRNA, and hashtag (#) indicates *P* < 0.05 compared to NT shRNA-transduced cells infected by same bacteria.

The canonical pathway by which pneumococcus and c-di-AMP stimulate IFNβ expression is via STING-mediated activation of IRF3 and IRF7 (29). The early phosphorylation of IRF3 and IRF7 was particularly triggered by infection with the *pde2* mutant (Figure 5A), implicating this pathway in the select responses of macrophages to these Δ*pde2* bacteria. To determine if STING was required for IFNβ induction by Pde2-deficient pneumococci, responses were compared between control RAW264.7 cells and STING-deficient RAW264.7 cells. The strong induction of IFNβ by Δ*pde2* mutants was abrogated by the absence of STING (Figure 5B). In contrast to IFNβ induction, cell death induced by pde2-deficient bacteria was not abrogated by STING deficiency (*P* = 0.15 from unpaired Student *t*-test on 3 independent experiments; 57 ± 6 and 51 ± 3 percent LDH after 24 h in STING-deficient and control RAW264.7 cells, respectively), dissociating these outcomes and suggesting that this bacteria-induced cell death may not require excessive IFNβ expression. The STING-dependent IFNβ induction was corroborated with independent experiments in which STING expression was knocked down by shRNA in human THP-1 cells. STING knock down inhibited IFNβ induction by the Δ*pde2* mutant pneumococci (Figure 5C). The STING-mediated induction of IFNβ stimulated by c-di-AMP requires cGAS (20). In parallel studies of human THP-1 macrophages in which cGAS was targeted by shRNA, induction of IFNβ mRNA by Δ*pde2* mutant pneumococci was reduced by cGAS knock down as effectively as the knock down of STING (Figure 5C). Altogether, these results implicate the cGAS-STING and IRF3/IRF7 pathways that mediate IFNβ induction by purified c-di-AMP (20) as underlying mechanisms of the distinctive responses of macrophages to Δ*pde2* mutant pneumococci.

### Dinucleotides Connecting the Phosphodiesterase Mutants to IFNβ Induction

**FIGURE 6:**
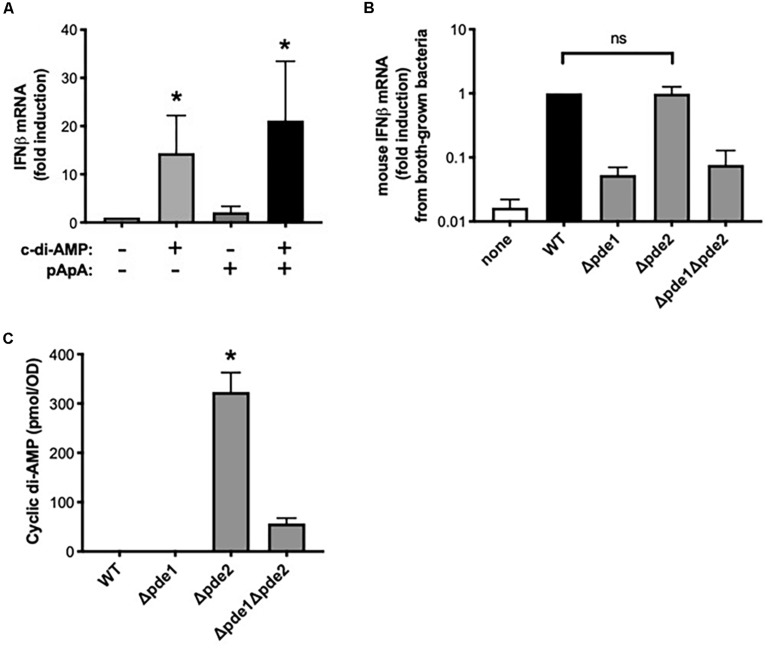
Dinucleotides involved in the distinctive effects of phosphodiesterase 2 mutant pneumococci. **(A)** Relative induction of IFNβ mRNA was measured in human THP-1 cell cultures that were matured to macrophage-like phenotypes using PMA before being stimulated 2 h with the indicated dinucleotides at a final concentration of 30 μg/ml. Human IFNβ mRNA was measured and normalized to 18S rRNA using qPCR, and expressed relative to the unstimulated cells (*N* = 3 experiments). Asterisk (*) indicates *P* < 0.05 compared to unstimulated cells. **(B)** Relative induction of IFNβ mRNA was measured in mouse macrophage-like RAW264.7 cell cultures infected 2 h with the indicated bacteria that were cllected after growth in roth culture instead of on blood agar plates. Mouse IFNβ mRNA was measured and normalized to 18S rRNA using qPCR, and expressed relative to the cells infected by WT bacteria (*N* = 3 experiments). **(C)** Relative c-di-AMP content was measured in pneumococcal cultures that were grown as used throughout experiments (except for in **B**), on inverted tryptic soy agar plates containing 5% sheep’s blood and in a 5% CO_2_ environment. Cyclic di-AMP was measured using a competitive ELISA (*N* = 4 experiments) and normalized to the bacterial density at a given optical density (OD). Asterisk (*) indicates *P* < 0.05 compared to other bacterial strains. “ns” indicates not significant (*P* > 0.05).

Although the pathways activated in macrophages by *pde2*-deficient pneumococci matched those induced by c-di-AMP, the Δ*pde2* mutant pneumococci were not expected to have higher levels of c-di-AMP than the other mutant strains, based on prior analyses (26). We considered whether Pde2 could have unique roles in bacteria that lead to different types of metabolites accumulating. Based on the different substrate specificities of the pneumococcal phosphodiesterases (26), the *pde2* deletion could result in the buildup of pApA in ways that the Δ*pde1* or Δ*pde1*Δ*pde2* mutants would not. To investigate whether excessive pApA could be a contributing factor to the burst of IFNβ expression in macrophages, we tested whether the pApA dinucleotide metabolite could stimulate macrophage IFNβ expression either alone or in combination with c-di-AMP. The pApA metabolite did not stimulate IFNβ expression at doses sufficient for c-di-AMP to do so (Figure 6A). Furthermore, no synergistic effects were seen when pApA was added to c-di-AMP (Figure 6A). These results do not support a role for pApA in either stimulating or exacerbating macrophage IFNβ expression.

Next, we considered whether the distinct responses of Δ*pde2* mutants could be due to unanticipated effects of phosphodiesterase mutations on c-di-AMP content in these bacteria. Previous studies of these pneumococcal strains reveal increased c-di-AMP in the Δ*pde1* and Δ*pde2* strains, with the greatest increase in the Δ*pde1Δpde2* double mutants (26). We assumed those effects of phosphodiesterase mutations would apply to bacteria in the present studies. However, the present studies involved bacteria grown on blood agar plates in humidified 5% CO_2_ environments, designed to enrich for virulence, whereas the prior studies were of bacteria grown in liquid broth and room air. Bacterial growth conditions can influence gene expression and metabolism (32, 33). Unlike with plate-grown bacteria, broth-grown Δ*pde2* mutants did not elicit a stronger induction of IFNβ mRNA compared to WT bacteria (Figure 6B), suggesting that mode of growth influences the phenotypes of bacteria and their interactions with macrophages. To investigate effects of the phosphodiesterase mutations on c-di-AMP content in bacteria as grown throughout the current studies (Figures 1–5), we measured relative c-di-AMP levels using an ELISA (34) in pneumococci that were grown on blood agar plates. Under these conditions, WT pneumococci did not have detectable c-di-AMP (Figure 6C). Similarly, the Δ*pde1* mutant grew with c-di-AMP levels remaining beneath the detection limit (Figure 6C). However, the Δ*pde2* and Δ*pde1Δpde2* double mutants accumulated c-di-AMP, with by far the greatest amounts in the Δ*pde2* single mutant bacteria (Figure 6C). These data suggest that growth conditions may have substantial effects on c-di-AMP metabolism, and that pneumococcal Pde2 has especially important roles in limiting c-di-AMP during the more biofilm-like and virulence-enhancing growth conditions used here. In conjunction with the prior data revealing distinct IFNβ responses from the Δ*pde2* mutant bacteria, these data strongly support the interpretation that macrophage responses are directly influenced by c-di-AMP levels in pneumococcus during infection.

## Discussion

This study suggests that the pneumococcal phosphodiesterase 2 enzyme has distinct and prominent roles in dictating bacterial c-di-AMP content and macrophage innate immunity. Deletion of the phosphodiesterase 2 gene resulted in multiple phenotypes that were not seen with other phosphodiesterase mutants, including pronounced c-di-AMP accumulation. In macrophages, the *pde2*-mutant pneumococci induced a rapid and strong induction of IFNβ and cytotoxicity, neither of which was observed after macrophage interactions with other pneumococci. These results were robust, observed *in vitro* with mouse and human cell lines as well as *in vivo* with primary murine alveolar macrophages. These data highlight a previously unappreciated complexity in how pneumococcal phosphodiesterase enzymes regulate c-di-AMP metabolism in bacteria and immunity in mammals.

It was unexpected that the *pde2*-mutant but not the *pde1*-mutant bacteria would substantially increase c-di-AMP levels in pneumococci grown as cultured here. These data suggest that when cultured on blood agar plates in a 5% CO_2_ environment, *pde2* is especially important for catabolizing c-di-AMP. It was further curious that the compound deletion of *pde2* with *pde1* had less pronounced effects, suggesting that other c-di-AMP regulatory mechanisms (24, 25) may compensate to diminish c-di-AMP levels when both of these 2 phosphodiesterase enzymes are absent from pneumococci. The present results with pneumococcus grown on solid media (a more biofilm-like condition) contrast with prior studies of these bacteria grown in a strictly planktonic liquid broth culture (26), suggesting that c-di-AMP metabolism varies across types of bacterial growth. This is plausible given the many roles c-di-AMP plays in bacterial physiology, including evidence that c-di-AMP itself helps determine planktonic vs. biofilm organization in streptococci (22, 35–38). Some virulence factors, like pneumolysin which can induce macrophage cytotoxicity as observed here, are more strongly expressed in biofilm-like growth states of pneumococci (32, 33). During infection, pneumococci transition between biofilm and planktonic phenotypes, and genetically identical pneumococci exhibit markedly different behaviors and transcriptomes in different anatomical sites involving planktonic or biofilm growth (33). In addition, there are different selection pressures on the pneumococcal phosphodiesterase enzymes that catabolize c-di-AMP across infected sites within a host, leading to the emergence of pneumococci with different phosphodiesterase gene mutations in different anatomic sites within infected individuals (39). Altogether, these data suggest strikingly dynamic roles of pneumococcal phosphodiesterases with impacts on c-di-AMP metabolism, pneumococcal phenotype, and infection that are highly context-dependent.

Macrophage signaling in response to c-di-AMP in the context of bacterial infection is incompletely understood. The steps involved in macrophage responses to extracellular cyclic dinucleotides (including c-di-AMP) are different from the pathways by which macrophages respond to cyclic dinucleotides delivered directly into the cytoplasm (20). Notably, extracellular cyclic dinucleotides require phagocytosis and phagosomal acidification before they can gain access to the cytosol (20). The membrane folate antiporter SLC19A1 mediates the transport of c-di-AMP across the membrane from phagosome lumen into cytosol (40). In the cytosol, c-di-AMP is recognized by a cGAS-STING complex which forms a signaling platform that leads to phosphorylation of IRF transcription factors (41). Our studies suggest similar pathways for c-di-AMP signaling during live bacterial infections. The triggering of IFNβ expression by *pde2*-mutant bacteria was prevented by an inhibitor of phagocytosis or of phagosome acidification, by cGAS knockdown, and by STING deficiency or knockdown. These results are consistent with a model in which macrophages ingest bacteria and bacteria-derived c-di-AMP into acidic phagosomes where the bacteria are degraded and c-di-AMP is released into the phagosomal lumen. Transport of c-di-AMP across the membrane into the cytosol then allows signaling via STING and cGAS to activate IRF3- and IRF7-mediated transcription of the gene for IFNβ.

The effects of *pde2* deletion on c-di-AMP metabolism in an *in vivo* infection would be multifactorial. Most importantly, the dysregulation of c-di-AMP metabolism due to phosphodiesterase deletion(s) can be disastrous for pneumococcal physiology, rendering them much more sensitive to diverse stresses including heat, osmotic shock, or UV radiation (26). They grow more slowly, exhibit altered morphology, and display reduced virulence (26, 42). Thus, such bacteria may be less fit or even incapable of effectively establishing infection. However, altered macrophage responses can also impact infection. Increased type I interferons can have complex effects during pneumococcal pneumonia. For example, type I IFN in pneumococcus infected lungs can induce *Sectm1* in lung epithelial cells to recruit neutrophils (43), which augments host defense but increases lung injury risk. Type I IFN signaling to the respiratory epithelium also helps to maintain a vigorous and resilient mucosal barrier against invasive pneumococcal disease (9–11). In contrast to this epithelial resilience, excessive IFNβ induced by *Salmonella* or inflammatory stimuli can kill macrophages by necroptosis (44, 45). In the present studies, the early IFNβ burst but not the later cell death triggered by Pde2-deficient pneumococci required STING, suggesting that excessive IFNβ might not be necessary for the cell death due to this strain of bacteria. Although the Pde2-deficient and c-di-AMP-rich pneumococci rapidly caused macrophage necrosis, the mode of cell death was not defined. Macrophage death can be detrimental during pneumonia in many ways (5). Across pneumococcal strains, those that more rapidly induce necroptosis tend to be more virulent in the lung (27), suggesting this mode of cell death to be a maladaptive pathway during pneumococcal pneumonia. In contrast, macrophage cell death by apoptosis appears to be a healthy response to pneumococcal infcetion, since specifically preventing macrophage apoptosis results in more severe lung infections (46). Pneumococcus-induced macrophage death is a complicated but important contributor to pneumonia outcomes. More research is needed to understand the natural variations in phosphodiesterase enzymes and c-di-AMP metabolism among pneumococci in the community and how these variations may influence cell death pathways including necroptosis, apoptosis, and more.

Finally, it was curious that all deletions of phosphodiesterase enzymes (*pde1*, *pde2*, or both) caused similar changes in macrophage NF-κB activity, including downstream cytokines like TNFα. Unlike the macrophage IRF phosphorylation, IFNβ induction, and cytotoxicity, macrophage NF-κB activity, and TNFα induction appear to dissociate from the bacterial c-di-AMP content. This could possibly result from non-canonical STING-dependent pathways through which DNA triggers NF-κB (47). These data suggest that some effects of phosphodiesterase enzymes in pneumococcus may be independent from their role in modulating c-di-AMP, implying novel substrates of pneumococcal phosphodiesterases yet to be identified.

The Pde2 enzyme appears to have distinct roles in c-di-AMP metabolism in pneumococci, which vary across growth conditions and perhaps infection settings. Loss of pneumococcal Pde2 function can profoundly elevate c-di-AMP and skew macrophage responses, including a dysregulated burst of IFNβ expression followed quickly by cell death. These results suggest that c-di-AMP in pneumococci is a pivotal factor driving innate immune responses in infection. Pneumococcal c-di-AMP may influence pneumonia and invasive pneumococcal disease. Additional studies should elucidate further the roles of phosphodiesterase 2 in bacterial physiology and host immune responses to pneumococcus.

## Materials and Methods

### Bacteria Strains and Growth Conditions

The strains of *S. pneumoniae* used here have been described in detail (23, 26). Unless otherwise specified, bacteria were grown for 10–15 h at 37°C in a 5% CO_2_ environment on inverted tryptic soy agar plates containing 5% sheep blood before being suspended in serum-free and antibiotic-free media to the desired optical density as a means of estimating concentration. For broth-grown bacteria, pneumococci were grown for 8 h in Todd Hewitt Broth with 0.5% Yeast extract (THY) as preculture, and preculture aliquots were used to seed planktonic cultures grown in THY for 6 h to ensure log-phase growth of all bacteria. Bacterial concentrations were initially estimated using optical density and then confirmed *post hoc* using serial dilution CFU assays.

### Cell Culture and Infection

For growth and maintenance, RAW264.7 cells (ATCC) were cultured at 37°C with 5% CO_2_ in Dulbecco’s Modified Eagle’s Medium (DMEM, Gibco) supplemented with 10% heat inactivated fetal bovine serum (Gibco), 100 IU of penicillin/mL, and 100 μg/mL of streptomycin. STING-deficient RAW264.7 cells (RAW-Lucia ISG-KO-STING cells; Invivogen) were maintained in the same media with Zeocin supplementation at 200 ug/mL. THP-1 Scrambled, STING knockdown, and cGAS knockdown cells were generated by transducing THP-1 cells with lentiviral vectors expressing shRNA targeting a scrambled sequence (48), STING (48), or cGAS (Sigma, TRCN0000149984), and maintained in RPMI 1640 (Gibco) supplemented with 10% heat inactivated fetal bovine serum (Gibco), 100 IU of penicillin/mL, 100 μg/mL of streptomycin, and 2ug/mL of puromycin, and incubated at 37°C with 5% CO_2_. For differentiation of THP-1 cells into a macrophage state, cells were resuspended in culture medium containing 100 nM phorbol myristate acetate (PMA, Sigma) for 24 h. Macrophages were lifted using Versene (Gibco) and replated for infection. For dinucleotide stimulation, pApA and c-di-AMP were purchased (InvivoGen) and added to PMA-matured THP-1 cells to a final concentration of 30 μg/ml. In infection experiments, cells were cultured in antibiotic-free media and *S. pneumoniae* were added at a dose of 10^7^ colony forming units (CFU) per well for 2 h at 37°C with 5% CO_2_. Indicated experiments included a 30-min preincubation with 2 μM cytochalasin D (Life Technologies), 200 nM bafilomycin A1 (Sigma), 50 μM chloroquine (Sigma), or 5 mM ammonium chloride (Sigma). None of the bacterial strains increased CFU/ml over 2 h in cell culture; while there was a possible trend for living Δ*pde2* and Δ*pde1Δpde2* mutants to decrease more than WT (suggesting poor stress responses), this did not reach statistical significance (*P* = 0.10 across groups in one-way ANOVA). For cell culture infection experiments lasting longer than 2 h, the antibiotic-free media was replaced with antibiotic-containing media after 2 h of culture with live bacteria. Studies with the NFκB-luciferase reporter RA264.7 cell line were performed as previously reported (27, 28).

### RNA Preparation and qRT-PCR

Total RNA was isolated with TRIzol reagent (Invitrogen) and Direct-zol RNA Kit (Zymo). qRT-PCR was performed using the RNA-to-Ct kit (Life Technologies) with the following primers and probes – mouse IFNβ: forward 5′-ACAGCCCTCTCCATCAACTATAA-3′, reverse 5′-CATCTT CTCCGTCATCTCCAT-3′, probe 5′-FAM-AGCTCCAGC/ZEN/TCCAAGAA-3′; mouse TNFα: forward 5′-TCATACCAG GAGAAAGTCAACCTC-3′, reverse 5′-TGGAAGACTCCT CCCAGGTATATG-3′, probe 5′-FAM-TGCCGTCAA/ZEN/GAGCCCCTG-3′; mouse IL-1β: forward 5′-GCCACCTTTTGA CAGTGATGA-3′, reverse 5′-GATGTGCTGCTGCCGAGA TT-3′, probe 5′-FAM-CTGCTTCCA/ZEN/AACCTTTGA-3′; mouse CXCL10: forward 5′-GGCCATAGGGAAGCTTGAA AT-3′, reverse 5′-GACATCTCTGCTCATCATTCTTT-3′, probe 5′-FAM-ATCGTGGCA/ZEN/ATGATCTCA-3′; mouse 18S ribosomal RNA: forward 5′-ATTCGAACGTCTGCCCTAT CA-3′, reverse 5′-GTCACCCGTGGTCACCATG-3′, probe 5′-FAM-TCGATGGTA/ZEN/GTCGCCGTG-3′. TaqMan Gene Expression Assays for human IFNβ were purchased from Applied Biosystems.

### Murine Model of Pneumococcal Pneumonia

C57BL/6 mice were purchased from Jackson Laboratory and housed in a pathogen free environment with access to food and water *ad libitum*. Mice were anesthetized using intraperitoneal injection of ketamine (50 mg/kg) and xylazine (5 mg/kg) and the trachea was surgically exposed. A 50 μL volume containing 10^7^ CFU of *S. pneumoniae* of specified strains (or saline vehicle as negative control) was intratracheally instilled via angiocatheter into the left lobe. Three hours after infection, mice were euthanized and the lungs were lavaged with 10 mL of phosphate buffered saline (PBS). Bronchoalveolar lavage cells were isolated by centrifugation and used as sources for RNA purification.

### Enzyme Linked Immunosorbent Assay (ELISA)

Cytokine levels of IFNβ were measured in a sandwich ELISA using rat anti-mouse IFNβ mAb (ab24324; Abcam) for capture, rabbit anti-mouse IFNβ polyclonal Ab (32400-1; PBL Biomedical Laboratories) for detection, HRP-conjugated donkey anti-rabbit IgG (711-036-152; Jackson ImmunoResearch Laboratories), and recombinant mouse IFNβ standard (12400-1; PBL Biomedical Laboratories). The detection limit of the IFNβ ELISA was 130 pg/ml. Cytokine levels of TNFα and IL-1β were determined using ELISA kits from R&D systems.

### Cell Death Assays

Cell death was measured by supernatant lactate dehydrogenase (LDH) using the Cytotox 96 Non-Radioactive Cytotoxicity Assay (Promega).

### Measurement of c-di-AMP

Bacterial strains were grown overnight on tryptic soy agar plates with 5% sheep blood at 37°C with 5% CO_2_. *S. pneumoniae* were suspended in DMEM to an OD_620_ of 0.4. After centrifugation of 6-ml aliquots, the bacterial pellets were resuspended in 500 μL 50 mM Tris–HCl pH 8.0 and lysed by sonication, followed by heating for 10 min at 95°C. Quantities of c-di-AMP were measured in the lysates using a competitive ELISA, as described (34). The amount of c-di-AMP was normalized to the bacterial density recorded at OD_620_.

### Statistics

Statistical analyses were performed using GraphPad Prism 8 (GraphPad Software). Data were presented as mean and standard error of the mean (SEM). All data were analyzed by one-way or two-way analysis of variance (ANOVA) with Sidak or Dunnett tests to correct for multiple comparisons. Values were log-transformed prior to analyses if they did not pass the F test for equal variance. Differences with *p* < 0.05 were considered statistically significant.

## Data Availability Statement

The datasets generated for this study are available on request to the corresponding author.

## Ethics Statement

The animal study was reviewed and approved by the Boston University Institutional Care and Use Committee.

## Author Contributions

AW, GB, and JM contributed to the conception and overall design of the study. GB generated mutant bacteria and performed c-di-AMP analyses. AW, AS, EA, and IM helped with designing, performing, and interpreting the individual experiments. HA and SG provided tools and insights relevant to STING and cGAS in human cells. AW performed statistical analyses. MJ, LQ, SG, GB, and JM helped with data analyses and interpretations. AW and JM wrote the first draft of the manuscript. JM managed the project. All authors provided help with the written manuscript including revisions and edits, and approved the submitted version.

## Conflict of Interest

The authors declare that the research was conducted in the absence of any commercial or financial relationships that could be construed as a potential conflict of interest.
